# Successful rituximab treatment of TAFRO syndrome with pathological findings of glomerular endothelial damage 

**DOI:** 10.5414/CNCS109400

**Published:** 2018-06-22

**Authors:** Yuhei Noda, Yosuke Saka, Akihisa Kato, Tetsushi Mimura, Tomohiko Naruse

**Affiliations:** Department of Nephrology, Kasugai Municipal Hospital, Kasugai, Japan

**Keywords:** TAFRO syndrome, Castleman disease, thrombotic microangiopathy, rituximab, ADAMTS13

## Abstract

Thrombocytopenia, anasarca, fever, renal insufficiency, and organomegaly constitute TAFRO syndrome, a variant of Castleman disease. We describe a patient with TAFRO syndrome who underwent renal biopsy. A 79-year-old woman was referred to us with fever and leg edema. She also had thrombocytopenia, pleural effusion, ascites, and acute kidney injury, and was admitted to our hospital. Her response to initial therapy with corticosteroid and cyclosporine was poor. Therefore, she received 4 doses of rituximab per week, which resulted in clinical improvement, including recovery of thrombocytopenia. A kidney biopsy thereafter showed diffuse, global glomerular endothelial injury indicating thrombotic microangiopathy (TMA). These findings suggested that TMA is associated with the thrombocytopenia and renal insufficiency of TAFRO syndrome.

## Introduction 

Several cases of TAFRO syndrome, which is characterized by thrombocytopenia, anasarca, fever, renal insufficiency, and organomegaly, have been reported as a variant of Castleman disease [1, 2, 3, 4, 5, 6, 7, 8]. The diagnostic criteria for TAFRO syndrome proposed by Masaki et al. [9] in 2015 include anasarca, thrombocytopenia, and systemic inflammation as major categories. TAFRO syndrome displays more acute clinical onset and less lymphadenopathy compared with Castleman disease. The mechanism and etiology of this syndrome remain unclear, especially associated renal insufficiency, which has not been pathologically studied in detail, although the pathological features of lymph nodes and bone marrow have been established. To the best of our knowledge, this is a rare case report of TAFRO syndrome with pathological findings of kidney damage. 

## Case report 

**Figure 1.**
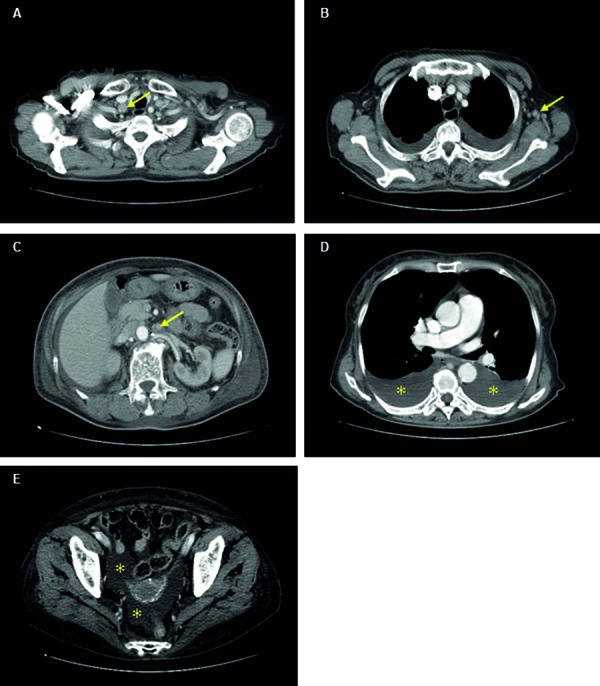
Radiographic findings. Whole body computed tomography showed multiple small lymphadenopathies (arrows) in the mediastinum (A), axilla (B), and para-aorta (C), bilateral pleural effusion (asterisks in D), and ascites (asterisks in E).

A 79-year-old woman with a medical history of type 2 diabetes mellitus, hyperlipidemia, and osteoporosis was referred to us with fever and leg edema. Her daily medications included glimepiride (1 mg), metformin (250 mg), olmesartan (10 mg), alfacalcidol (0.5 µg), and pitavastatin (2 mg). She had no medical history of severe hypertension. Her physical findings were unremarkable except for bilateral pitting edema of both lower extremities, a body temperature of 37.8 °C, and blood pressure of 177/105 mmHg. Although she did not receive ophthalmological examination, symptomatic visual impairment was not observed. Blood pressure was improved without additional medication after admission. Whole body computed tomography revealed multiple small lymphadenopathies in the mediastinum, axilla, and para-aorta, bilateral pleural effusion, and ascites (Figure 1). Laboratory findings revealed severe thrombocytopenia (0.9 × 10^4^/µL), along with elevated serum creatinine (1.85 mg/dL) and C-reactive protein (CRP 3.90 mg/dL). Serum IL-6 and vascular endothelial growth factor values were not elevated at 3.76 (normal range, 0.45 – 9.96) and 15.6 (normal range 0.0 – 38.3) pg/mL, respectively. ADAMTS13 (a disintegrin-like and metalloproteinase with thrombospondin type 1 motifs 13) activity was decreased to 34.4%, which was not low enough to be shown in typical TTP (thrombotic thrombocytopenic purpura). The ADAMTS13 inhibitor was not detected. Urinalysis revealed proteinuria (2.65 g/g creatinine) and microscopic hematuria (1 – 4 red blood cells per high-power field) (Table 1). Bone-marrow aspiration was a dry tap, which indicated bone marrow fibrosis. Lymph nodes were not biopsied due to the absence of palpable lymph nodes and severe thrombocytopenia. 

Hemodialysis was initiated on hospital day 7 to treat the acute kidney damage. Initial pulse therapy with methylprednisolone (mPSL; 500 mg/day for 3 days) followed by prednisolone (PSL; 40 mg/day) did not improve the thrombocytopenia, anasarca, and kidney damage. Plasma exchange was started on day 11 and continued for a total of three sessions, but a clinical response was not achieved. We diagnosed TAFRO syndrome based on the refractory anasarca, thrombocytopenia, and elevated CRP. 

Intravenous rituximab (500 mg/week for four weeks) started on hospital day 30 dramatically improved the thrombocytopenia, anasarca, and kidney damage. Hemodialysis was discontinued on hospital day 57. 

On day 79, after thrombocytopenia had resolved, five glomeruli were obtained via kidney biopsy. Pathological findings determined by light microscopy comprised the diffuse and global duplication of basement membranes and mesangiolysis. Significant changes in arterioles, tubules, and interstitium were not found. Electron microscopy revealed subendothelial swelling of glomerular capillaries (Figure 1). Immunofluorescence was negative for IgG, IgA, IgM κ/λ, C3, C1q, and C4. 

**Figure 2. Figure2:**
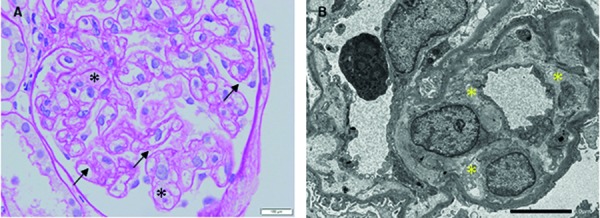
Pathological renal findings. A: Light microscopy image shows diffuse and global duplication of basement membranes (arrows) and mesangiolysis (asterisks). Periodic acid-Schiff stain; original magnification, × 400). B: Electron microscopy image shows subendothelial swelling of glomerular capillaries (asterisks).

Three months after rituximab therapy, the thrombocytopenia, anasarca, and kidney damage did not recur while on low-dose corticosteroid therapy (PSL 5 mg/day). She was discharged from our hospital on day 159. After remission, ADAMTS13 activity was increased to 73.6%, whereas the ADAMTS13 inhibitor remained undetected. Corticosteroid therapy was discontinued after 3 months from discharge. Clinical remission has been maintained during 9 months from the discontinuation of corticosteroid therapy. [Fig Figure2]

## Discussion 

Thrombocytopenia, anasarca, fever, renal insufficiency, and organomegaly are features of TAFRO syndrome, which is a multicentric variant of Castleman disease [3]. Several clinicopathologic differences between the two pathologies have recently been discussed, and Masaki et al. [9] proposed diagnostic criteria, a disease severity classification, and a treatment strategy for TAFRO syndrome in 2015. The findings of our patient met all the major and two minor categories of the proposed diagnostic criteria. All lymphadenopathies in this patient were notably < 1.5 cm in diameter, which also met the diagnostic criteria, and the score on the proposed disease severity classification was 10, which corresponds to “severe”. 

Previous reports indicate that various agents including cyclosporine [6], tocilizumab [4, 5], and rituximab [2, 8, 10] have been used to treat TAFRO syndrome. We assumed that our patient would not respond to tocilizumab because her serum IL-6 value was normal. Therefore, we selected rituximab for additional therapy. Indeed, the serum IL-6 level is lower in TAFRO syndrome than in Castleman disease, which might explain why some previously documented patients with TAFRO syndrome were quite refractory to tocilizumab [6, 8]. 

The pathological renal findings of light and electron microscopy indicating TMA represent the key strength of the present report. Several studies have investigated renal involvement in Castleman disease [11, 12], whereas TAFRO syndrome with pathologically confirmed renal TMA has been rarely documented as far as we can ascertain [13, 14, 15]. Although the mechanism remains unclear, TMA might be associated with thrombocytopenia and renal insufficiency in TAFRO syndrome. Pathological findings of previous reports [13, 14, 15] are similar to those of this case, which support our speculation. In this case, ADAMTS13 activity recovered after remission. Fujiwara et al. reported IL-6 involvement in ADAMTS13 activity [4]. Of interest, other factors might be involved in this case, because the serum IL-6 value of this case was not increased. In addition, a good response to rituximab indicates that humoral factors secreted by B cells might function in TMA in cases of TAFRO syndrome. 

In conclusion, this is a rare description of TAFRO syndrome with pathological renal findings. More pathological renal data are required to clarify the etiology of TAFRO syndrome. 

## Funding 

This study received no grant support. 

## Conflict of interest 

The authors declare no conflict of interest. 

**Table 1.** Laboratory findings.

WBC	8,100/µL	AST	18 IU/L	ASO	×40	HBs Ag	(–)
Neu	85%	ALT	7 IU/L	ASK	29 IU/mL	HCV Ab	(–)
Lym	9%	LDH	272 IU/L	C3	85.0 mg/dL	HIV Ab	(–)
Eos	0%	ALP	223 IU/L	C4	23.5 mg/dL	HTLV-1 Ab	(–)
Mono	5%	γ-GTP	16 IU/L	IgG	1,834 mg/dL	HHV-8	< 4.0 × 10 copy
Hb	8.9 g/dL	T-Bil	0.8 mg/dL	IgA	312 mg/dL	anti-HP Ab	(–)
MCV	100.0 fl	TP	6.3 mg/dL	IgM	74 mg/dL	IL-6	3.76 pg/mL
MCH	32.7 pg	Alb	2.4 mg/dL	IgG4	49 mg/dL	VEGF	15.6 pg/mL
Plt	0.9×10^4^/µL	BUN	33.7 mg/dL	ANA	×40	ADAMTS13	
		Cre	1.85 mg/dL	pattern	Speckled	activity	34.4%
PT-INR	1.04	UA	8.3 mg/dL	anti-dsDNA	(–)	inhibitor	(–)
APTT	32.7 sec	Na	136 mEq/L	anti-Sm	(–)		
Fib	400 mg/dL	K	3.8 mEq/L	MPO-ANCA	(–)	Urinalysis	
D-dimer	6.4 µg/mL	Cl	105 mEq/L	PR3-ANCA	(–)	sugar	(+2)
		Ca	8.1 mg/dL	Coombs		protein	(+2)
BS	188 mg/dL	P	4.5 mg/dL	direct	(+)	occult-blood	(+2)
HbA1c (NGSP)	6.9%	CRP	3.90 mg/dL	indirect	(–)	UPCR	2.65 g/gCr
		PCT	4.61 ng/mL	PA-IgG	70.2 ng/10^7^cells	RBC	1 – 4/HF
		Ferritin	175 ng/mL	s-IL2R	1,137 U/mL	WBC	20 – 29/HF
		Haptoglobin	136 mg/dL				

WBC = white blood cells; Neu = neutrophils; Lym = lymphocytes; Eos = eosinophils; Mono = monocytes; Hb = hemoglobin; MCV = mean corpuscular volume; MCH = mean corpuscular hemoglobin; Plt = platelets; PT-INR = international normalized ratio of prothrombin time; APTT = activated partial thromboplastin time; Fib = fibrinogen; BS = blood sugar; AST = aspartate transferase; ALT = alanine transaminase; LDH = lactate dehydrogenase; ALP = alkaline phosphatase; γ-GTP = γ-gluta-myltranspeptidase; T-Bil = total bilirubin; TP = total protein; Alb = albumin; BUN = blood urea nitrogen; Cre = creatinine; UA = UA uric acid; CRP = C-reactive protein; PCT = procalcitonin; ASO = anti-streptolysin O antibody; ASK = anti-streptokinase antibody; ANA = anti-nuclear antibody; anti-dsDNA = anti-double stranded DNA antibody; anti-Sm = anti-Smith antibody; MPO-ANCA = myeloperoxidase anti-neutrophil cytoplasmic antibody; PR3-ANCA = proteinase 3 anti-neutrophil cytoplasmic antibody; PA-IgG = platelet-associated IgG; s-IL2R = soluble interleukin-2 receptor; HTLV-1 = Ab human T cell leukemia virus type 1 antibody; HHV-8 = human herpesvirus-8; anti-HP Ab = anti-helicobacter pylori antibody; IL-6 = interleukin-6; VEGF = vascular endothelial growth factor; ADAMTS13 = a disintegrin-like and metalloproteinase with thrombospondin type 1 motifs 13; UPCR = urine protein-to-creatinine ratio; RBC = red blood cell.
